# Family involvement in managing medications of older patients across transitions of care: a systematic review

**DOI:** 10.1186/s12877-019-1102-6

**Published:** 2019-03-29

**Authors:** Elizabeth Manias, Tracey Bucknall, Carmel Hughes, Christine Jorm, Robyn Woodward-Kron

**Affiliations:** 1 Centre for Quality and Patient Safety Research, School of Nursing and Midwifery, Deakin University, 221 Burwood Highway, Burwood, VIC 3125 Australia; 2 Alfred Health, Commercial Road, Prahran, VIC 3181 Australia; 3 School of Pharmacy, Queen’s University Belfast, University Road, Belfast, Northern Ireland BT7 1NN UK; 4 Sydney Medical School, The University of Sydney, Edward Ford Building A27, Fisher Road, Camperdown, NSW 2050 Australia; 5 NSW Regional Health Partners, 72 Watt St, Newcastle, NSW 2300 Australia; 6 School of Medicine, The University of Melbourne, Grattan Street, Parkville, VIC 3052 Australia

**Keywords:** Transitions of care, Family, Medication management, Older patients, Family involvement, Hospitals, Home, Aged care facilities

## Abstract

**Background:**

As older patients’ health care needs become more complex, they often experience challenges with managing medications across transitions of care. Families play a major role in older patients’ lives. To date, there has been no review of the role of families in older people’s medication management at transitions of care. This systematic review aimed to examine family involvement in managing older patients’ medications across transitions of care.

**Methods:**

Five databases were searched for quantitative, qualitative and mixed methods empirical studies involving families of patients aged 65 years and older: Cumulative Index to Nursing and Allied Health Literature Complete, Medline, the Cochrane Central Register of Controlled Trials, PsycINFO, and EMBASE. All authors participated independently in conducting data selection, extraction and quality assessment using the Mixed Methods Appraisal Tool. A descriptive synthesis and thematic analysis were undertaken of included papers.

**Results:**

Twenty-three papers were included, comprising 17 qualitative studies, 5 quantitative studies and one mixed methods study. Families participated in information giving and receiving, decision making, managing medication complexity, and supportive interventions in regard to managing medications for older patients across transitions of care. However, health professionals tended not to acknowledge the medication activities performed by families. While families actively engaged with older patients in strategies to ensure safe medication management, communication about medication plans of care across transitions tended to be haphazard and disorganised, and there was a lack of shared decision making between families and health professionals. In managing medication complexity across transitions of care, family members perceived a lack of tailoring of medication plans for patients’ needs, and believed they had to display perseverance to have their views heard by health professionals.

**Conclusions:**

Greater efforts are needed by health professionals in strengthening involvement of families in medication management at transitions of care, through designated family meetings, clinical bedside handovers, ward rounds, and admission and discharge consultations. Future work is needed on evaluating targeted strategies relating to family members’ contribution to managing medications at transitions of care, with outcomes directed on family understanding of medication changes and their input in preventing and identifying medication-related problems.

**Electronic supplementary material:**

The online version of this article (10.1186/s12877-019-1102-6) contains supplementary material, which is available to authorized users.

## Background

As older patients’ needs develop increased complexity, they are more likely to have changes in their health that require treatment with medications. When older patients become acutely ill, hospitalisation may be required, which necessitates medication management across transitions of care. ‘Transitions of care’ refers to the times when patients transfer between settings of care, such as hospitals, home, rehabilitation care and long-term care, between locations or within the same location, including admission and discharge [1]. Medication errors are likely to occur at transitions of care because of the potential for communication breakdown during activities such as bedside handovers, ward rounds, and admission and discharge consultations between health professionals, older patients and families [2]. Previous research has shown that medication error rates associated with transitions between hospitals, residential aged care facilities and home vary between 19 and 80% [3, 4].

Many medication management activities are carried out by families of older patients. These activities include: assisting with administering medications, recognising therapeutic benefits and adverse effects of medications, and clarifying information for patients [5, 6]. To date, there has been no review of the role of families in older people’s medication management at transitions of care. Therefore, the aim of this systematic review was to examine family involvement in managing older patients’ medications across transitions of care. A specific mnemonic for systematic reviews, PICo, was used to develop the research question. The components for the research question according to PICo are population (families), phenomenon of interest (managing older patients’ medications) and context (transitions of care) [7]. The research question that guided the systematic review is: how are families involved in managing older patients’ medications across transitions of care?

## Methods

A systemic review was undertaken of research studies using a best practice guide for conducting systematic reviews [8].

### Eligibility criteria

Inclusion criteria comprised research of any design – qualitative, quantitative and mixed methods – involving families of older patients aged 65 years and older. Families were defined as formal relations of older patients or other significant individuals who played an important role in the older people’s lives. Research had to involve older people moving between different settings. Papers were still considered if medication management was not the central focus of the study but was identified within the findings. Papers not published in English were excluded.

### Information sources

The literature search was conducted in the following electronic bibliographic databases from inception to end December 2017: The Cumulative Index to Nursing and Allied Health Literature (CINAHL) Complete (Elton B. Stephens Co Host (Ebscohost)), Medical Literature Analysis and Retrieval System Online (MEDLINE) (Ebscohost), the Cochrane Central Register of Controlled Trials (CENTRAL) (The Cochrane Library), Psychological Information Database (PsycINFO) (Ebscohost), and Excerpta Medica Database (EMBASE) (refer to Additional file 1 for Medline search) [9]. Hand searching of reference lists was also conducted for relevant studies. Cochrane systematic reviews were searched to locate relevant papers. However, reviews themselves were not included in the final dataset. The grey literature was also searched using Google Scholar to locate other original, peer-reviewed research.

### Search and study selection

The following key terms, and variations thereof, were searched as four separate groups of terms: (1) family, carers, caregivers, and relatives; (2) older patients, older people, and older adults, geriatric, seniors, elderly; (3) medication, medicines, medication management and medicines management; and (4) admission, discharge, transfer, transition, transitions of care, and transition points. These terms were subsequently combined. One author completed the search with assistance from the university research librarian, and all authors independently determined the eligibility of retrieved papers for inclusion at the abstract and full text levels. The authors comprised individuals with different perspectives and discipline expertise. It was therefore perceived there was value in each author independently checking the literature to minimise selection bias, and to improve the rigor of the study selection process. There were a number of studies identified at the initial search that investigated family involvement at transitions of care. At the full text level, unless there was some mention in the results of families’ contribution in older patients’ medications, a particular study was excluded.

### Data extraction and evaluation

Information was extracted from each paper on the type of study conducted, and the settings in which each study was undertaken. Information was also noted on the data collection processes used, and the patients and families who participated. To prepare the data for synthesis, qualitative and quantitative data located within the results section of papers were extracted and incorporated into a spreadsheet.

Each paper was independently assessed by two reviewers using the Mixed Methods Appraisal Tool (MMAT), which provided a quality score for qualitative, quantitative and mixed-method studies [10]. Any discrepancies were discussed until consensus was reached. No studies were excluded because of the quality score.

### Data synthesis

Data synthesis of qualitative data was achieved using a thematic approach. These data were read and re-read to increase familiarity and understanding with the content. Line-by-line coding was undertaken using words and phrases within and across studies. Words and phrases were grouped together, which were clustered into categories. These categories were further examined for identification of themes and subthemes [11].

In synthesising quantitative results, it was not possible to undertake meta-analysis due to the heterogeneity of outcomes and variability in operational definitions. Therefore, a descriptive synthesis was conducted of the major findings. These quantitative studies were also examined to determine how the results fitted into the themes and subthemes generated from qualitative data. All quantitative data that were transferred to a spreadsheet were subsequently re-written and transformed into narrative forms to describe and explain the results. These rewritten narrative forms were read several times and examined to determine how they could be identified as categories. These categories were compared and contrasted with other categories to determine how they could be grouped into already developed themes and subthemes, or whether they could be grouped into new themes and subthemes. All authors scrutinised the content and structure of themes and subthemes, and the ways in which studies of different research designs were represented and mapped within these themes and subthemes.

By means of an example, the quantitative results of the Towle et al. paper [12] were integrated into themes in the following way. The results were rewritten in a descriptive way to explain the impact of an evidence-based quality improvement initiative to enhance patient and family preparedness in care transition. The following details were documented. After implementation of the quality improvement initiative, health professionals interacted with patients and families in goal-directed ways to convey information. Subsequently, family members’ understanding had improved of the patients’ medical condition, medications prescribed, treatment plan and follow up care. These results mapped onto the theme: Giving information and receiving feedback, and the subtheme: health professionals informing families. The results also mapped onto the theme: Managing medication complexity across transitions of care and the subtheme: supporting family participation in interventions. Mixed methods studies were handled according to the approaches described for both qualitative and quantitative data. Only information from the results section of included studies was used for synthesis and no information was used from the discussion section.

## Results

**Fig. 1:**
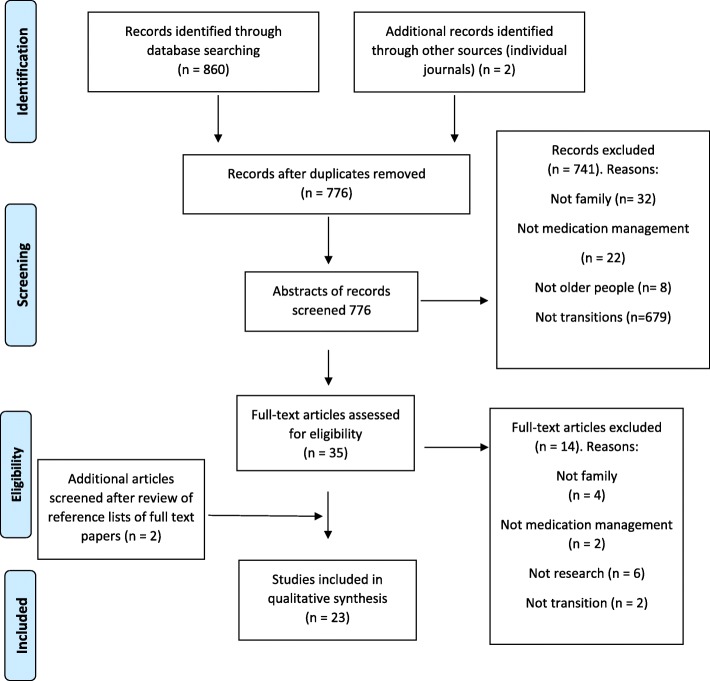
Flowchart for determining included papers

In all, 860 papers were identified through database searching. An additional two papers were added following a manual screen of full text papers. A total of 23 papers were eligible for inclusion (Fig. 1).

Qualitative exploratory designs comprised data collection methods involving semi-structured interviews (*n* = 11), observations (*n* = 2), diaries and interviews (n = 2), and focus groups and interviews (n = 2). Quantitative designs included two randomised controlled trials, one quasi-experimental study, two cross-sectional survey studies, and one mixed method study comprising interviews and an electronic medical record review. MMAT findings showed that 15 studies obtained a score of 75%, which meant that three-quarters of the criteria were met, and 6 had a score of 50%, which meant that half of the criteria were met (Table 1).

**Table 1:** Characteristics of included papers examining family involvement for managing medications of older patients across transitions of care (*N* = 23)

Author, year, country, MMAT result	Design and setting	Description of study	Participants	Key Findings
Allen et al. 2017 [13] Australia***	Design: Qualitative exploratory. Setting: metropolitan public health-care network	Data collection: interviews. Transition points involved: acute ward setting and rehabilitation setting in hospital, home. Medication management process involved: admission instruction, discharge instruction.	Patients *n* = 19. 78.9 years (age range 45–94 years for patients and families). Gender: not stated. Family *n* = 7. Gender: not stated.	Caring relationships with health professionals: - Nurses in rehabilitation attended to follow-up phone calls to check on discharge management. - Lack of continuity of medical practitioners and interactions with multiple medical practitioners. - Medical decision making about discharge medications without understanding about medications prescribed by other medical practitioners. Seeking information: - If patients was too unwell to seek information during their acute illness, family wanted to have medication information on their behalf. - Expectation that doctors would share medication information with them during the hospital admission. This did not always occur. - Family members were well informed about changes to medications in rehabilitation ward setting. - General practitioner (GP) relied on accurate and timely discharge summary to explain medication information to family.
Coleman et al. 2006 [15] United States**	Design: Randomised controlled trial. Setting: one hospital, eight skilled nursing facilities, one home health care agency.	Data collection: rates of rehospitalisation measured at 30, 90, and 180 days. Transition points involved: community; skilled nursing facility; rehospitalisation. Medication management processes involved: medication self-management. Intervention – four pillars. - Support patients and family with medication self-management, medication reconciliation. - Patient-centred record to assist with site transitions. - Timely follow-up with care. - Supply patients and family with list of “red flags” for worsening condition.	Patients: *n* = 379 intervention group, *n* = 371 control group. Age for intervention group: mean 76.0 years (SD 7.1 years). Age for control group: mean 76.4 years (SD 6.8 years). Gender: 48% female for intervention group. 52% female for control group. Medical condition: at least 1 of 11 selected acute or chronic conditions. Family recruited with patients: n = not specified. Relationship with patient: not specified.	Outcomes: Rehospitalisation rates at 30 days – Intervention group = 8.3% Control group = 11.9%, *p* = 0.048. Rehospitalisation rates at 90 days – Intervention group = 16.7% Control group = 22.5%, *p* = 0.040. Rehospitalisation rates for the same condition that precipitated the index hospitalisation at 90 days – Intervention group = 5.3% Control group = 9.8%, *p* = 0.04. Rehospitalisation rates for the same condition that precipitated the index hospitalisation at 180 days - Intervention patients: 8.6% Control patients: 13.9%, *p* = 0.046. Mean hospital costs at 180 days - Intervention patients: $2058 Control patients: $2546, log-transformed *p* = 0.049.
Coleman et al. 2004 [14] United States***	Design: Quasi experimental study with intervention and control groups. Setting: one community, hospital.	Data collection: rates of rehospitalisation at 30, 90 and 180 days; care transition measure Transition points involved: hospital, community residence facility, home. Medication management process involved: medication self-management. Intervention – four pillars. - Support patients and family with medication self-management, medication reconciliation. - Patient-centred record to assist with site transitions. - Timely follow-up with care. - Supply patients and family with list of “red flags” for worsening condition. Intervention involved: meetings with transitions coach; communication tool (personalised medical record, role plays), follow up phone calls.	Patients: *n* = 158 intervention group, *n* = 1235 control group from administrative data. Age for intervention group: mean 75.1 years (SD 6.4 years). Age for control group: mean 78.5 years (SD 7.5 years). 54% female for intervention group. 55% female for control group. Medical condition: at least one or more of 9 medical conditions. Family recruited with patients: n = not specified. Relationship with patient: not specified.	Outcomes: Rehospitalisation at 30 days – Intervention group compared with control group: 0.52 (95% confidence interval (CI) 0.25, 0.96) Rehospitalisation at 90 days – Intervention group compared with control group: 0.57 (95% CI 0.25, 0.72) Rehospitalisation at 180 days – Intervention group compared with control group: 0.57 (95% CI 0.36, 0.92). Intervention group - patients reported: high level of confidence in obtaining information, communicating with health care team, and understanding their medication regimen. Quality of care transitions taxonomy – Intervention group: 9.5% of posthospital transitions were complicated. Control group: 14.9% of transitions, *P* = 0.35.
Crawford et al. 2015 [16] Australia***	Design: Qualitative, exploratory. Setting: one aged rehabilitation and geriatric evaluation and management facility	Data collection: Semi-structured interviews. Transition points involved: acute hospital setting, aged rehabilitation and geriatric evaluation and management facility Medication management process involved: discharge planning.	Family *n* = 20. Relationship with patient: husbands, wives, daughters, son, daughter-in-law, close friends of people with dementia.	Themes: Adjusting to new role from caregiver to visitor – - Difficulty in relinquishing role. - Desire to continue to be involved in decisions. - Felt ongoing responsibility to communicate medication needs. - Belief about specialised knowledge about relative’s care. - Staying informed helped caregivers to cope with move to facility.
Deeks et al. (2016) [17] Australia**	Design: Qualitative, exploratory. Setting: three urban primary and acute sites, one rural primary and acute site.	Data collection: semi-structured interviews. Medication management process involved: hospital admission and discharge for patients with dementia.	51 participants comprising doctors, nurses, pharmacists, occupational therapists, general practitioners, Alzheimer’s Australia staff, and family. Family n = not specified. Relationship with patient: not specified.	Themes: Medication reconciliation – - Verifying accurate list on admission was difficult if family members not present or medications not brought to hospital. Lack of modified planning for care transitions – - Lack of identification that patient had cognitive problems as they moved across settings. - Inadequate information about medication changes at discharge. - Carers’ support for use of dose administration aids by patients, but potential problems with errors. Lack of assessment of patients’ ability to use aid. - Desire for once-daily dosing if paid carer required to visit at home after discharge. Multiple prescribers – - Little information shared between private and public hospitals. - Treatment delays between specialist and general practitioner as communication by letter. Residential aged care facilities – - Lack of accurate and complete information from hospital. Medication reviews by pharmacists – - Patients with dementia trusted and built rapport with community pharmacists and general practitioners. Patients not receptive to home medicine reviews.
Dyrstad et al. (2015) [18] Norway**	Design: Qualitative, exploratory. Setting: two hospitals	Data collection: observations, conversations, observational field notes. Transition points involved: two emergency departments (EDs), seven hospital wards; including admissions from home based care or nursing homes, and discharge. Medication management process involved: admission instruction, discharge instruction.	Patients *n* = 41. Age: mean 86.0 years, range 73–97 years. Gender: 46% female. Medical condition: an orthopaedic diagnosis or chronic condition, and poly-pharmacy (> 5 medications daily). Family *n* = 28. Relationship with patient: son, daughter, wife. Health professionals n = not specified. Disciplines involved: paramedics, nurses, doctors.	Themes: Information dissemination and decision-making – - No scheduled discharge planning meetings with patient and family. - Nurse phoned family to inform them of doctors’ decisions. Next of kin important advocates – - In ED, provided valuable information about medications taken before admission. - Family administered medications in ED during busy times. - In wards, no routines to invite family to participate on doctor’s rounds. - Informed on day of discharge about ward round decisions. - Some families had to seek information about medication decisions at discharge.
Georgiadis & Corrigan 2017 [19] United Kingdom***	Design: Phenomenological. Setting: three hospitals.	Data collection: Interview, audio diary, written diary. Transition points involved: clinical settings (not described), home. Medication management process involved: medication counselling at discharge.	18 participants Patients *n* = 12. Age: 65.9 years (SD 17.2 years). Gender: Mixed, not stated. Medical condition: not mentioned. Patients with non-medical complex conditions, which were not defined. Family *n* = 6. Relationship with patient: not specified.	Themes: Limited involvement in discharge-care preparations – - Premature discharges meant lack of planning in medication counselling. - Unexpected and delayed discharges meant that family unable to be present. Weak service interface – - Expected organisation of appointments for outpatient clinic or home visits did not happen. Reliance on family for medication administration. - Family arranged primary clinic to assess medications.
Hagedoorn et al. 2017 [20] The Netherlands***	Design: Qualitative exploratory. Setting: Four general hospitals,	Data collection: non-participant observations, audio-recordings of planned discussions for admission and discharge discussions and family meetings. Transition points involved: admission to ward, 13 clinical wards comprising neurology, pulmonary, internal medicine, cardiology, geriatrics. Discharge from ward. Medication management process involved: administration, monitoring.	Patients *n* = 62. Age: 76 years (SD 7.2 years). Gender: 48% female. Medical condition: 22 patients (36%) had 3 or more chronic diseases. Family *n* = 107 at planned discussions. Relationship with patient: husband, wife, son, daughter.	Themes: Social network support – - Support by family caregivers assisting with or monitoring medication intake at home. Not addressed by nurses in hospital assessment sessions. Coordination of care – - During discharge discussions nurses reviewed home medication list with patients and family. - Family asked specific questions about changes in the patients’ home medications.
Hvalvik & Reierson, 2015 [21] Norway***	Design: Phenomenological hermeneutic design. Setting: hospital.	Data collection: in-depth interviews. Transition points involved: hospital, municipal rehabilitation, short-term care facility, home. Medication management process involved: discharge planning, self-management at home.	Family *n* = 11. Relationship with patient: son, spouse, daughter.	Themes: Balancing vulnerability and strength – - Enduring emotional stress with discharge. - Family expected to be involved in discharge, but were not included. - Worry about being discharged too early and lack of medication information. - Lack of communication about medications during stay. - Sense of responsibility in managing medications. - Lack of communication between hospital and community services. Prescription of medications that should have been ceased due to adverse effects or allergies. Coping with an altered everyday life – - Dealing with changes to family routines. - Anticipating possible problems if patients discharged early. - Comprehensive understanding about older person’s vulnerability, and fragility.
Jeffs et al. 2017 Canada***	Design: Qualitative exploratory. Setting: orthopedic inpatient units in two acute care hospitals and one orthopedic unit at a complex continuing care rehabilitation hospital.	Data collection: semi-structured interviews. Medication management process involved: care involving transfer from an acute care hospital to a rehabilitation hospital.	Patients *n* = 13. Age: 82.9 years (range: 68–91 years). Gender: 69% female. Medical condition: non-elective patients who had fallen or sustained a fracture though an accident. Family *n* = 9. Relationship with patient: child, spouse, partner, sibling. Health professionals *n* = 50.	Themes: Watching – - Alert to medication administration and changes in patients’ status. - Patients’ lack of understanding about medication changes. Being an active care provider – - Responsibility for providing care that health care providers performed. - Administering medication was easier for family to do than the nurses, as nurses experienced challenges with the patient taking medication. - Lack of engagement by health care providers about involving family. Advocating – - Being supportive of patients’ needs such as changing medication times to suit patients’ routines. Navigating the health care system – - Asking questions and coordinating follow-up care. - Arranging appointments with various members of interdisciplinary team. - Lack of availability of health care providers to ask questions about transitions plan.
King et al. 2013 [23] United States***	Design: Qualitative study using grounded dimensional Analysis. Setting: five non-profit religious and government skilled nursing facilities (SNF)	Data collection: focus group and individual interviews Transition points involved: skilled nursing facilities, hospital. Medication management process involved: hospital discharge.	Health professionals n = 27. Disciplines involved: nurses.	Themes: Reconciling hospital information- - Seeking medication details from families was problematic as sometimes not adequately informed about family members contact details. - Asking families about medication details created a poor first impression of SNF staff. Consequences of poor-quality discharge communication – - Care delays and implementation of an inappropriate medication plan. - Inaccurate hospital information produced family dissatisfaction. - Made the SNF appear unorganised.
Knight et al. 2013 [24] United Kingdom**	Design: Qualitative exploratory. Setting: participants’ home	Data collection: semi-structured interviews, medication diary. Transition points involved: hospital, home Medication management process involved: discharge process.	Patients n = 7. Age: > 75 years. Gender: 43% female. Medical condition: not stated. Family n = 12. Relationship with patient: wife, husband.	Themes: Discharge in general – - Long delays till discharge or abrupt notification about discharge. Obtaining medication for discharge- - Waiting for medications to be prepared at hospital pharmacy. Information regarding discharge medication – - Carer belief that it was their responsibility to check understanding on individual medicines. - Carer satisfaction about information provided but not detailed. - Medication changes in hospital not conveyed to carers. - Inadequate explanations of new medicines and associated risks for the patient. Medication lists – - Lack of written guide available or had never received a list. Communication about medication in hospital and following discharge – - Carers detected medication omissions after careful examination. - Needed to feel better prepared with medications post-discharge.
Lowson et al. 2012 United Kingdom***	Design: Qualitative exploratory. Setting: participants’ home	Data collection: semi-structured interviews. Transition points involved: hospital, home. Medication management process involved: medication information at admission.	Patients n = 27. Age: mean = 79.0 years (SD 4.25 years). Gender: 52% female. Medical condition: heart failure or cancer in the last year of life. Family n = 12. Relationship with patient: wife, husband, daughter, sister, neighbour, friend.	Themes: Conductors – - Strong contributions to maintaining good care throughout illness trajectory. - Detailed knowledge about medications. - Alerted health professionals about potential medication errors. Second fiddle – - Following hospital admission, ability to work with health professionals to influence decisions vastly reduced. - Carers invested effort in maintaining continuity of relationship. - Advocated on patients’ behalf to affect beneficial change.
Nazarath et al. 2001 United Kingdom***	Design: Randomised controlled trial. Setting: three general hospitals, one long-stay hospital	Data collection: Transition points involved: general hospital wards, home. Medication management process involved: discharge information. Intervention: (for intervention studies) Discharge plans developed by pharmacists, home visit by community pharmacist, counselling patients and family on appropriate doses and purpose. Control group: discharge letter to general practitioner.	Patients: n = 181 intervention group, *n* = 181 control group. Age for intervention group: mean 84 years (SD 5.2 years). Age for control group: mean 84 years (SD 5.4 years). Gender: 62% female for intervention group. 66% female for control group. Medical condition: had a mean of three chronic conditions. Family (n = not specified) Included in intervention but not stated.	Outcomes: Hospital readmission at 3 months – Intervention group: 64 (39%) Control group: 69 (39.2%), *p* > 0.05. Hospital readmission at 6 months – Intervention group: 38 (27.9%) Control group: 43 (28.4%), *p* > 0.05 No differences between groups: Patients’ general well-being, satisfaction with the service and knowledge of and adherence to prescribed medication (p > 0.05).
Neiterman et al. 2015 [25] Canada**	Design: Qualitative exploratory. Setting: participants’ home	Data collection: interviews Transition points involved: hospital, home Medication management process involved: medication management at home	Patients *n* = 17. Age: 70–89 years, mean = 79 years. Gender: 41% female. Medical condition: diverse chronic illnesses. Family *n* = 19. Relationship with patient: husband, wife, mother, father, daughter, son, daughter-in-law, son-in-law.	Themes: Dealing with medical confusion – - Post-discharge medication management was difficult because of medication changes. Facilitators for recovery: social capital and social support - - Family members made sure that medications schedules were followed. - Family was overwhelmed and exhausted due to constant need to coordinate care and ensure patients’ needs met. Targeted nurse practitioner initiative - - Some caregivers did not fully understand the nurse practitioner (NP) role but relied on NPs to oversee the management of medications.
Palagyi et al. 2016 [26] Australia***	Design: Qualitative exploratory. Setting: three long-term care facilities	Data collection: Focus groups and interviews Transition points involved: long-term care facilities, hospital, community care. Medication management process involved: deprescribing medications.	Patients *n* = 25. Age: mean = 87.6 years, range 75–100. Gender: 77% female. Medical condition: not stated. Family *n* = 16. Relationship with patient: not mentioned. Health professionals *n* = 27. Disciplines involved: general practitioners, long-term care facilities staff.	Themes: Pitfalls of coordinated care - - Lack of review of acute medication after condition was treated. Negotiating a complex system - - Family concerned that compulsory two-year residential medication management review schedule was too long. Many changes occur in two years. Medication knowledge - - Family were unfamiliar with specific medication indications.- Minimal recognition of adverse drug reactions. Whatever the GP says goes - - Complete trust in the care and decisions of the GP. - Number of specialists involved in residents’ care. Need for realistic expectations- - Relatives viewed long-term care facilities as active providers of medical care. GPs regarded it as a palliative care environment.
Ploeg et al. 2017 [31] Canada***	Design: Interpretive descriptive. Setting: participants’ home	Data collection: semi-structured interviews. Transition points involved: home, primary care settings, hospital. Medication management process involved: managing medications for multiple comorbidities.	Patients n = 41. Age: 17% aged 85 years and over. Gender: 44% female. Medical condition: three or more chronic conditions. Family *n* = 47. Relationship with patient: wife, husband, grandfather, mother-in-law, friends. Health professionals *n* = 42. Disciplines involved: registered nurse, registered/licensed practical nurse, personal support worker/healthcare aide	Themes: Experience of managing multiple chronic conditions - - Emotionally draining. Organising pills and appointments - - Managing changes to medications that frequently occurred after an acute care hospitalization. - Abrupt discharge, without input from caregivers about preferences. - Organising appointments to discuss blood results and scans affecting medications. Being split – - Receiving services from multiple providers who focus on a single disease - Lack of communication between family doctor and specialists. Doing what the doctor says - - Family believed decision making was physician-directed. Providers perceived their approach involved shared decision-making.
Popejoy 2011 [32] United States***	Design: Qualitative exploratory. Setting: tertiary care hospital	Data collection: semi-structured interviews. Transition points involved: hospital, home, residential aged care facility. Medication management process involved: Discharge planning.	Patients n = 13. Age: mean = 84 years, range = 72–89 years. Gender: 62% female. Medical condition: no cognitive impairment, at least pre-clinically frail. Family n = 12. Relationship with patient: spouses. Health professionals n = 7. Disciplines involved: registered nurses, social workers.	Themes: Changing the discharge plan - *-* Patients and caregivers were adamant about going home (not to residential care). - Health professionals were worried when family had trouble understanding about medications. - Family were sometimes old with health problems themselves, and had difficulties coping with and remembering to offer patients’ medications at home.
Tjia et al. 2014 [27] United States***	Design: qualitative exploratory. Setting: Three hospice agencies.	Data collection: semi-structured interviews Transition points involved: hospice, outpatient oncology, primary care settings. Medication management process involved: medication prescribing across transitions.	Patients *n* = 18. Age: mean = 80 years (SD 10 years). Gender: 42% female. Medical condition: advanced cancer. Family *n* = 8. Relationship with patient: not stated. Health professionals *n* = 17. Disciplines involved: nurses, physicians.	Themes: Medication coordination and communication - - Families were keen to have comprehensive medication reviews upon transition to hospice that assessed ongoing use of longstanding medications for comorbid illness. - Family were receptive to reducing harmful and non-essential medications.
Towle et al. 2012 [12] Singapore*	Design: Prospective observational, cross-sectional. Setting: one tertiary hospital	Data collection: survey questionnaire Transition points involved: hospital, home. Medication management process involved: Discharge process. Intervention: (for intervention studies) BOOST (Better Outcome for Older adults through Safe Transitions); an evidence-based quality improvement initiative to enhance care transition in improving patient/family preparedness for discharge.	Patients *n* = 40. Age: not stated. Gender: not stated. Medical condition: > 1 chronic condition. Family n = not stated. Relationship with patient: not stated.	Outcomes: Patient and caregiver understanding of medical condition - - Improvement by 70%. Patient and caregiver understanding of medications- - Improvement by 67%. Patient and caregiver understanding of treatment plan - - Improvement by 81%.Patient and caregiver understanding of follow-up - - Improvement by 41%.
Trollor 1997 [28] Australia****	Design: Cross-sectional. Setting: Community palliative care service.	Data collection: questionnaire. Transition points involved: palliative care services, home, primary care services. Medication management process involved: symptom management.	Patients n = 26. Age: not stated. Gender: 31% female. Medical condition: patients with palliative care needs. Family *n* = 26. Relationship with patient: wives, daughters, husbands.	Themes: - 13 out of 26 family members were in charge of patients’ medication. - Role in dealing with general practitioners and palliative care specialists, to administer medication for pain, sleeping difficulty and loss of appetite were most challenging.
White et al. 2015 [29] United States***	Design: Qualitative exploratory. Setting: Two hospitals.	Data collection: semi-structured interviews. Transition points involved: hospital, home. Medication management process involved: Discharge planning.	Patients n = 20. Age: mean = 77 years (SD 8.8 years). Gender: 47% female. Medical condition: stroke survivors. Family n = 9. Relationship with patient: husband, wife, son, daughter.	Themes: Preparing to go home after the stroke - - Importance of health professionals understanding family’s needs following discharge so that specific situation could be addressed. - Wrong family member targeted for providing information. Complexity of medication management- - some family were confident about their knowledge. - Some family lacked understanding about the purpose of medication. - Usually family member who managed medications. - Difficulties in managing medications for patients with swallowing problems. Random decisions about which medications should be crushed.
White et al. 2014 [33] United States**	Design: Mixed methods. Setting: Two hospitals.	Data collection: semi-structured interviews, electronic medical record review. Transition points involved: hospital, home. Medication management process involved: Discharge planning.	Patients *n* = 310. Age: mean = 76 years (SD 9.8 years). Gender: Not stated. Medical condition: stroke survivors. Family *n* = 20 combined with patients. Relationship with patient: not stated.	Themes: Within one month of discharge, 10% were readmitted and 25% within 6 months. Reasons for readmission were recurrent stroke/transient ischaemic attack (19%), pneumonia and urinary tract infection (19%), swallowing problems and dehydration (9%), and cardiac causes (7%). - Need for guidance on what to expect at home. - Need follow-up in community about early identification of problems. - Complexity of medication management sometimes led to lack of understanding.

Note: The symbols *, **, *** and **** refer to scores of 25, 50, 75 and 100% respectively obtained on the Mixed Methods Appraisal Tool (MMAT)

Four themes were identified: giving information and receiving feedback, participating in decision making, managing medication complexity across transitions of care, and supporting family participation in interventions (Table 2).

**Table 2:** Synthesised themes from included studies

Themes and subthemes	Studies with the first named author and year																						
	Allen 2017	Coleman. 2006	Coleman 2004	Crawford 2015	Deeks. 2016	Dyrstad 2015	Georgiadis 2017	Hagedoorn 2017	Hvalvik 2015	Jeffs 2017	King 2013	Knight 2013	Lowson 2012	Nazarath 2001	Neiterman 2015	Palagyi 2016	Ploeg 2017	Popejoy 2011	Tjia 2014	Towle 2012	Trollor 1997	White 2015	White 2014
Giving information and receiving feedback																							
Families conveying information to health professionals	X			X		X			X	X	X	X	X		X	X					X	X	
Health professionals informing families	X	X	X		X	X	X	X	X	X	X	X		X					X	X		X	
Participating in decision making																							
Medication decisions occurring on admission						X																	
Medication decisions occurring transfers and discharge	X			X	X	X	X	X	X			X			X		X						
Medication decisions occurring after discharge				X				X			X		X			X	X						
Characteristics of health professionals and families	X								X				X				X	X					
Managing medication complexity across transitions of care																							
Challenges in managing medication complexity from hospital admission to discharge										X			X					X					
Difficulties affecting medication complexity in the community and aged care facilities	X						X		X		X	X	X		X	X	X	X				X	X
Possibilities for individualised tailored care					X				X				X				X						
Supporting family participation in interventions		X	X											X						X			

### Giving information and receiving feedback

Eighteen studies addressed the theme of giving information and receiving feedback [12–29]. There were two subthemes relating to this theme: families conveying information to health professionals about patients’ medication-taking behaviour and activities, and health professionals informing families about medication changes.

Families played a crucial role in information giving during patient admission to hospital, when patients with dementia moved to long term care [16], for patients receiving palliative care [28] or in their last year of life [30] and when patients moved from hospital to home [13, 18, 21, 24, 25, 29]. Family members perceived that their role was as ‘knowledge keepers’ about patients’ medications, particularly those with chronic conditions [16, 18]. Whilst some family members indicated that they understood how to administer medications, they were less clear about how these medications worked and their potential adverse effects, especially for patients who had experienced a recent critical event, such as a stroke [26, 29].

In patients who lacked cognitive capacity such as those with dementia, hospital doctors indicated that it was difficult for them to obtain and verify a complete and accurate list of current medications, and to address the discrepancies between the medication list and medications actually consumed by patients [17]. In these situations, hospital pharmacists perceived that verification of the medication list on admission was dependent on family members. If family members were not present, or they did not bring the patients’ medications to the hospital on admission, hospital pharmacists indicated that their responsibility was to attempt to contact general practitioners or community pharmacists [17]. Family members perceived themselves as being able to effectively provide medication information at crucial times on behalf of patients, such as those experiencing delirium or those with confusion following a femur fracture [18], and to clarify patients’ expectations to health professionals, thereby leading to reduced patient anxiety [22].

Some family members considered that misunderstandings about medications related to their own lack of initiative rather than to the responsibility of health professionals [24]. In contrast, other family members indicated that they played the role of “conductors” (p. 1197) of information for older patients in the last year of life [30]. In this role, family members had a regular presence, provided detailed knowledge about patients’ medication-taking activities, and alerted health professionals about potential medication errors [13, 30].

Keeping families informed about medication changes was considered important. Family members stated they were concerned about doctors notifying patients about medication changes in situations where patients were compromised due to alterations in their physical and psychological status [13, 22]. Family members felt frustrated if doctors did not convey medication changes to them [13, 16, 23, 24], and family members valued information provided by pharmacists upon discharge from hospital [13, 24] and by general practitioners in relaying medication changes after discharge [13]. Conversely, family members reported that nurses were too busy to provide medication information [17, 21, 24]. Families also valued receiving written medication guides or medication lists as practical ways of keeping them informed; however, these resources were not always available [24]. Sometimes, community nurses provided information to the ‘wrong’ family members, which then had to be redirected to others [29].

### Participating in decision making

This theme related to how families participated in decision making with, or on behalf of, patients in managing medications across transitions of care. Fourteen studies examined this theme [13, 16–21, 23–26, 30–32]. Four subthemes underpinned this theme: medication decisions occurring on admission to hospital, those occurring during transfers and at discharge, those that happened after discharge home and to aged care facilities, and characteristics of health professionals and family members in fostering participation in decision making.

In one participant observation study in emergency departments, family members made decisions about administering medications [18]. They determined the specific times to give medications to patients. This occurred at busy times while patients waited to be transferred to other settings. An example included a daughter providing anti-epileptic medications to her mother because of understaffing and heavy nursing workloads in the emergency department [18].

Many challenges impeded family participation in decision making during transfers and at discharge. Unexpected or delayed transfers and discharges due to staff constraints of doctors, nurses and pharmacists resulting in insufficient time and planning, led to limited opportunities for families to participate in medication decisions [19, 20, 24, 25, 31]. Family members believed that some health professionals, such as community nurses or specialists focused on treating a single condition, that health professionals did not listen to them, and that health professionals organised discharge medications without understanding about patients’ concerns and the home situation that could impact on their treatment regimen [13, 16, 31]. There was lack of family participation in medication decisions in diverse communication encounters, including informal bedside conversations, ward rounds, and discharge consultations [17–19]. The absence of structured routines for family communication meant that family members were not present at times when goals of care were discussed [18]. For example, family members complained about nurses contacting them by phone to inform them about medication decisions on the day of discharge, with these decisions having been made much earlier during the patients’ hospital stay. Family members preferred that doctors and nurses had a routine of contacting them about participating on the doctors’ rounds relating to planning for discharge. [18].

Planning activities for patient transfer or discharge often lacked an individualised approach, as shown by the absence of dedicated sessions to discuss goals of treatment [17, 19]. Some assertive family members questioned nurses about how patients would use medications to deal with worsening symptoms, such as difficulties in breathing or increasing confusion [20], and the effects of changed medications at the time of discharge [17]. Nevertheless, opportunities for their involvement tended to be more apparent if nurses organised specific family meetings where family members could ask questions [20]. In patients with dementia who had changing demands for managing behavioural and psychological symptoms, transitioning between various environments was common [16]. Their families believed that they played a vital role in making decisions at home; however, their roles blurred and shifted between being an active caregiver and a passive bystander as patients transferred from home, to acute care, rehabilitation and geriatric evaluation and management units, and back to home [16]. Family member sought to be called by health professionals to enable pre-planning to organise a time to discuss about medications.

Lack of involvement during transitions contributed to safety concerns about medications [19, 20, 24, 25, 31]. Families were given little time to plan for assisting with medication administration at home [20] and they were confused about the changes made, which meant they did not know what to do when patients went home [24, 25, 31]. Family members commented that they did not feel involved in decisions because they received inadequate explanations for medication changes [24, 31]. Family members’ lack of participation sometimes led to increased risk, such as prescribing medications for patients where allergies existed and were not clarified [31].

Following discharge, family members participated in decisions by assisting with, and monitoring medication intake for, patients at home. In examining patients’ medication regimens, community nurses rarely discussed the patients’ home situation [20]. At home, family members perceived themselves as advocating for patients’ needs, by changing medication times to suit patients’ individualised routines [22], and by reminding health professionals about the medication schedules followed at home [25]. For patients discharged home after receiving palliative care services, 13 out of 26 family members indicated that they took charge of patients’ medications at home, to manage symptoms such as pain, sleeping difficulty and loss of appetite [28]. Conversely, for residents situated in long-term care facilities, care workers tended to exclude family members from decision making. In these environments, some family members experienced difficulties in relinquishing their carer role and wanted to retain their role in decisions [16]. Family members of patients discharged to aged care facilities were frustrated about their lack of inclusion in decisions made at the acute care hospitals from where the patients were transferred. They observed aged care nurses making multiple requests to the acute care hospitals, seeking clarification of unclear discharge orders for patients discharged to aged care services [23]. Furthermore, family members felt uncomfortable in questioning general practitioners about the medication plans they prepared for individuals situated in residential aged care facilities [26].

Interpersonal characteristics of health professionals and family members affected how and whether decision making took place. Family members commented that if nurses and doctors exuded a positive attitude and explained their role, this approach facilitated family participation [13]. Some health professionals were perceived to lack empathy, with an inability to actively listen to family concerns about medications. Pharmacists and doctors believed it was their role to consider the complexities of managing older patients’ multiple medications. However, these health professionals felt challenged in addressing the risk-benefit concerns in following individual guidelines and trying to tailor medication regimens for older patients with several health conditions [31]. Family members often felt a sense of responsibility for managing patients’ medications [21]. Patients delegated this responsibility to family members because they did not want to be perceived as bothersome and complaining to clinical staff [30]. Health professionals sometimes changed their belief that family members would be able to cope with managing patients’ medications when they sought information from family members [32].

### Managing medication complexity across transitions of care

There were 14 studies that focused on managing medication complexity across transitions of care [13, 17, 19, 21–26, 29–33]. Medication complexity referred to rapidly changing dose and frequency orders, medications prescribed to treat several co-existing conditions, and complicated administration and storage requirements. Three subthemes related to this theme: challenges in managing medication complexity from hospital admission to discharge, difficulties affecting medication complexity in the community and aged care facilities, and possibilities for individualised tailored care for managing medications.

In managing medication complexity from hospital admission to discharge, families experienced difficulties in understanding how the health system operated in hospitals [21], while at the same time, they felt responsible for supporting patients’ complex needs. Families described playing a “second fiddle” role (p. 1197) during hospital admission of patients living with heart failure or lung cancer in their final year of life, where families felt subordinate to doctors and the hospital system [30]. Families considered that health professionals perceived them as outsiders, and they had to be assertive to have their views heard [22].

Hospital discharge meetings were organised in an attempt to help patients and families to understand patients’ complex treatment regimens [13, 19, 22]. However, families perceived that discharge activities were not tailored to patients’ complex needs. Quick discharge processes were organised for patients with multiple chronic conditions [31] and planning was not modified to take account of patients with cognitive problems [17].

Family members also had a poor first impression of skilled nursing facilities (a form of aged care facility in the United States), when health providers asked them about medications prescribed after patients’ transfer from hospital to these facilities. Instead, family members believed that health professionals in hospitals should provide clarification about these prescriptions at the point of transfer [23]. In this particular circumstance, family members felt it was wrong that they were expected to provide this clarification.

Several studies reported difficulties in administering complex medication regimens in the community and aged care facilities [17, 21, 25, 32]. For pharmacists offering dose administration aids to patients at discharge, there was often insufficient screening to determine the appropriateness of using these aids at home. Older patients needed to have a reasonable degree of cognition in order to work out the timing of doses, and to push out medications from the aids [17]. Interviews with nurses working in a skilled nursing facility identified poor communication and inappropriate medication plans as patients moved from the hospital to the facility. Nurses believed that health professionals in hospitals did not utilise families effectively to obtain information during transfers. Complicated medication changes at discharge created disruptions to daily routines at home for patients and families [25] and since families were unable to understand the rationale for some changes, they did not remember to remind patients about when to consume these medications [32]. Families preferred patients to have once-daily doses of medications, especially for those patients with multiple medications as paid carers often only visited patients once a day. However, this preference was not usually considered by prescribers [17]. Families also found themselves frequently reminding home nurses about medication orders that these nurses should have stopped due to a patient’s history of allergies or adverse events because of medications leading to vomiting and confusion [21].

For patients needing home palliative care services, families were challenged in dealing with unresolved symptoms of pain, sleeplessness and loss of appetite, along with the diagnosis of cancer. As these patients’ symptoms worsened, constant changes had to be made to medication orders, thereby adding difficulties for families [28]. Similarly, families wanted patients to have a compulsory medication review by hospice nurses in hospice environments to assess ongoing need for medications used for long-standing conditions. However, hospice nurses perceived doctors were reluctant to discontinue medications in these patients, and that these doctors lacked confidence in making medication assessments [27]. Furthermore, for older people moving into Australian residential aged care facilities, families believed that 2 years was too long to wait for a planned formal medication review [26]. There was also concern about acute conditions being treated for months after conditions had subsided, including antihistamines and antibiotics [26]. At the same time, families were uncertain about what medications that doctors should prescribe following a patient’s stroke compared to what they prescribed prior to a stroke, and when patients had multiple comorbidities, such as hypertension and Parkinson’s disease [29, 33]. Families also had to contend with multiple prescribers who managed patients’ conditions [17, 31]. Prescribers often focused on single disease states and therefore did not always consider the effects of their prescriptions on other chronic conditions or medications being taken [21].

Medication changes created the challenge of new side-effects requiring additional monitoring, which were sometimes not adequately conveyed to family members [31]. For example, in the study by White et al. [29] involving patients following a stroke with impaired swallowing, families did not receive clear information from doctors about which medications could be crushed.

Several studies considered possibilities for individualised tailored care, which was more likely when health professionals delayed patient discharge to provide more time for coordinating medication regimens [19, 21, 30, 31]. Proactive approaches used between family members, such as maintaining regular contact with each other, enabled continuity of care for patients with heart failure or cancer in the last year of life [30]. Such approaches enabled greater family understanding of how medication changes occurred as patients’ condition altered [21, 31]. However, there was little focus of how patients’ demographic characteristics such as those from disadvantaged or vulnerable backgrounds could affect tailored care for managing medication regimens.

### Supporting family participation in interventions

Four studies focused on supporting family participation, together with that of patients, in interventions aimed at improving patients’ experiences at transitions of care [12, 14, 15, 34]. In two papers by Coleman and colleagues [14, 15], an intervention was delivered by an advanced practice nurse to patients and families that included medication self-management, a patient-centred record, primary care and specialist follow-up, and knowledge of warning signs or symptoms that indicated a worsening condition. This intervention was designed to enhance patient and family self-management skills across transitions of care. To provide support with medication self-management, the advanced practice nurse reviewed each medication with the patient, as well as the family member if available, to ensure that the patient understood its purpose, instructions, and potential adverse effects. Aside from identifying that family members were involved as recipients of the intervention, no details were provided of how they perceived these interventions, how they were specifically involved as a separate or collaborative entity to patients, and how their involvement influenced outcomes. In both studies, there were significant reductions in rehospitalisation rates and costs after discharge from the initial hospitalisation (*p* < 0.05). No outcomes relating to medication errors were reported.

In the intervention study by Nazarath et al. [34], discharge plans were developed by hospital pharmacists, home visits occurred with community pharmacists, and counselling took place with patients and family on appropriate doses and purpose of medications. No differences were found between control and intervention groups in terms of readmission at 3 months and 6 months, or in patients’ well-being, satisfaction with the service, and knowledge of and adherence to prescribed medications (*p* > 0.05). There was no specific investigation of how family involvement may have influenced the clinical outcomes.

In Towle et al.’s [12] prospective observational study, the focus was on evaluating the effectiveness of BOOST (Better Outcome for Older adults through Safe Transitions) in improving transition from hospital to home. After delivery of the intervention, comprising a bundle of evidence-based tools on discharge processes, the authors reported patients’ and families’ improved understandings of medications by 67%, of the treatment plan by 81%, and follow-up by 41%. No details were provided of the families’ specific contributions in achieving these outcomes.

## Discussion

Four major themes were identified in this systematic review: information giving and receiving, participation in decision making, managing medication complexity, and family participation in supportive interventions. In the studies identified, families’ involvement in medication management tended to be subsumed in various aspects of patients’ care. It was therefore sometimes difficult to identify discrete details about the family perspective on medication management.

Information giving was a key area of family involvement, which tended to occur in restricted ways. Health professionals acknowledged family members as key sources of information about the medications that older people were prescribed. However, the process of eliciting information from family members largely focused on patient admission to hospital [18, 20]. There was also some indication of information seeking from family members for older patients with dementia who moved from acute care settings to long term care [16] and of older patients at their last year of life from hospital to home [30]. No evidence was found with regard to information seeking from family members as older patients moved between various settings within hospitals. As health professionals mainly sought information from families on patient admission, this creates the possibility of medication discrepancies at future transitions of care. Information giving needs to occur in diverse environments, including when older patients move to home or to residential aged care facilities.

Receiving information by family members from health professionals was a relatively disorganised and haphazard process [17–19]. There was little evidence of families receiving information from health professionals if older patients moved between different acute care settings within hospitals, or when movements occurred from acute care to subacute care or long-term care settings. Information receipt that focused on the time of discharge from hospital to home, sometimes created a management burden as health professionals attempted to provide medication counselling and education at a single time point. Medication errors were therefore a possibility following discharge. Rather than focusing information receiving at discharge, it may be more effective if this activity occurs throughout the patients’ hospital stay. Similarly, efforts in information giving and receiving could be reorganised such that different approaches are available to deal with differing levels of patient and family understanding.

Participation in decision making demonstrated that families communicated with patients about strategies to use when taking medications as they moved from one environment to another. In some environments where health professionals were affected by time constraints, understaffing, and heavy workloads, there was sometimes little opportunity for health professionals to attend to older patients’ medication needs [18]. In these situations, families were recipients rather than active participants of decisions [35]. Greater attention should be placed on enabling shared decision making during planned communication encounters including family meetings, bedside handovers, ward rounds, and admission and discharge consultations.

In managing medication complexity across transitions of care, family members perceived a lack of tailoring of medication plans for patients’ needs, and believed they had to display perseverance to have their views heard. In studies that dealt with medication complexity, there was insufficient demographic information to indicate whether families had disadvantaged or vulnerable circumstances, such as being of non-English speaking backgrounds, low socioeconomic groups, or low health literacy. Improving understanding about medication complexity at transitions of care requires fundamental changes in the way that health professionals and families interact with each other. Some past work has focused on the use of patient teach-back to address misinterpretations about the medication regimen [36, 37]. As an extension of that process, family teach-back could be an effective means of identifying key information whereby family members use their own words to explain to health professionals what they know about patients’ medications. Individuals can then collectively verify understandings, address misconceptions and improve family and health professional comprehension [38].

Four interventions were identified as being directed to patients and families; however, the specific contribution of families was not clearly distinguished [12, 14, 15, 34]. Improvements in family understanding about medications were identified in one intervention study [12] while in another, no improvements were found in medication knowledge or understanding [34]. The remaining two studies had no information about medication management outcomes [14, 15]. While families were named as recipients of interventions, there was insufficient clarity about the extent of their involvement, and of how their involvement affected outcomes achieved. No details were provided about compliance with interventions, or difficulties encountered with family involvement. To address gaps in past work, targeted strategies need to focus more specifically on family members’ active contribution to managing medications at transitions of care, with outcomes directed on family understanding of medication changes and their input in preventing and identifying medication-related problems.

There are limitations associated with the systematic review. Since the review only included papers published in English, it is possible that those published in other languages may have provided further insights on the topic. As most papers did not specifically focus on the objective of this systematic review, it was sometimes difficult to extract the required information. There was also no attempt to exclude papers on the basis of quality.

There were a number of methodological issues relating to the included studies. In terms of strengths, of the studies included in the review, 65% had obtained a MMAT score of 75%, thereby indicating the majority of the studies were relatively well-conducted. Interview studies showed that the data were rich, comprehensive, and revealed clear insights into the contextual challenges affecting older patients across transitions of care. In relation to methodological limitations, transitions of care tended to be examined at only particular time points, namely, movements of patients during admission to or discharge from hospital. Given that the focus of the work involves family involvement, it is interesting that only two studies comprised qualitative observational designs.

The systematic review indicates the need for further research. Future studies should focus on examining families’ contribution and involvement in managing older patients’ medications across transitions of care. Most past work generally only addressed family involvement in general aspects of patient movement across transitions of care. Greater consideration needs to be given to family involvement in the continuum of patient journey at different contexts of care, from admission to hospital, through to transfers across wards and across hospitals, and discharge home or to residential aged care facilities.

## Conclusions

Families play an important role in supporting older patients in managing their medications as these patients move across different settings. Nevertheless, there is lack of acknowledgement from health professionals of the activities performed by families before, during and following these movements. While families actively engage with older patients in strategies to ensure medication safety, further work is needed on measuring the effectiveness on these strategies on medication outcomes, facilitating shared decision making between families and health professionals, and clarifying medication plans of care across transitions.

## Additional file

Additional file 1: Search History/Alerts Medline (Ebscohost) [9]. Search history for Medline. (DOCX 54 kb)

## Abbreviations

BOOST: Better Outcome for Older adults through Safe Transitions
CENTRAL: Cochrane Central Register of Controlled Trials
CINAHL: Cumulative Index to Nursing and Allied Health Literature
Ebscohost: Elton B. Stephens Co Host
EMBASE: Excerpta Medica Database
MEDLINE: Medical Literature Analysis and Retrieval System Online
MMAT: Mixed Methods Appraisal Tool
PICo: population, phenomenon of interest and context
PsycINFO: Psychological Information Database
