# Supplementary material for: Family involvement in managing medications of older patients across transitions of care: a systematic review
Source: BMC Geriatr. 2019 Mar 29;19:95. doi: 10.1186/s12877-019-1102-6 (PMC6441224; doi:10.1186/s12877-019-1102-6)
Supplement: Supplementary file 1 — Search History/Alerts Medline (Ebscohost) [9]. Search history for Medline. (DOCX 54 kb) [file 12877_2019_1102_MOESM1_ESM.docx]

**Search History/Alerts Medline (Ebscohost) [9]**

- [Print Search History](javascript:__doPostBack('ctl00$ctl00$FindField$FindField$historyControl$ctl00$PrintHistoryLink',''))
- [Retrieve Searches](javascript:__doPostBack('ctl00$ctl00$FindField$FindField$historyControl$ctl00$RetrieveSearchesLink',''))

- [Retrieve Alerts](javascript:__doPostBack('ctl00$ctl00$FindField$FindField$historyControl$ctl00$RetrieveAlertsLink',''))

- [Save Searches / Alerts](javascript:__doPostBack('ctl00$ctl00$FindField$FindField$historyControl$ctl00$SaveSearchesLink',''))

 Select / deselect all  

|  | [Search ID#](javascript:__doPostBack('ctl00$ctl00$FindField$FindField$historyControl$ReorderHistoryLink','')) | **Search Terms** | **Search Options** | **Actions** |
| --- | --- | --- | --- | --- |
|  | S26 | S21 AND S22 AND S23 AND S24 | **Limiters** - Date of Publication: 19810101-20171231  **Search modes** - Boolean/Phrase | [**View Results**](javascript:__doPostBack('ctl00$ctl00$FindField$FindField$historyControl$HistoryRepeater$ctl00$linkResults','')) (426)  [**View Details**](javascript:showShDetails(%22ctl00_ctl00_FindField_FindField_historyControl_ctrlPopup%22,%20%22S26%22);)  [**Edit**](http://web.b.ebscohost.com/Legacy/Views/UserControls/Ehost/) |
|  | S25 | S21 AND S22 AND S23 AND S24 | **Search modes** - Boolean/Phrase | [**View Results**](javascript:__doPostBack('ctl00$ctl00$FindField$FindField$historyControl$HistoryRepeater$ctl01$linkResults','')) (466)  [**View Details**](javascript:showShDetails(%22ctl00_ctl00_FindField_FindField_historyControl_ctrlPopup%22,%20%22S25%22);)  [**Edit**](http://web.b.ebscohost.com/Legacy/Views/UserControls/Ehost/) |
|  | S24 | S15 OR S16 OR S17 OR S18 OR S19 OR S20 | **Search modes** - Boolean/Phrase | [**View Results**](javascript:__doPostBack('ctl00$ctl00$FindField$FindField$historyControl$HistoryRepeater$ctl02$linkResults','')) (1,178,824)  [**View Details**](javascript:showShDetails(%22ctl00_ctl00_FindField_FindField_historyControl_ctrlPopup%22,%20%22S24%22);)  [**Edit**](http://web.b.ebscohost.com/Legacy/Views/UserControls/Ehost/) |
|  | S23 | S11 OR S12 OR S13 OR S14 | **Search modes** - Boolean/Phrase | [**View Results**](javascript:__doPostBack('ctl00$ctl00$FindField$FindField$historyControl$HistoryRepeater$ctl03$linkResults','')) (358,567)  [**View Details**](javascript:showShDetails(%22ctl00_ctl00_FindField_FindField_historyControl_ctrlPopup%22,%20%22S23%22);)  [**Edit**](http://web.b.ebscohost.com/Legacy/Views/UserControls/Ehost/) |
|  | S22 | S5 OR S6 OR S7 OR S8 OR S9 OR S10 | **Search modes** - Boolean/Phrase | [**View Results**](javascript:__doPostBack('ctl00$ctl00$FindField$FindField$historyControl$HistoryRepeater$ctl04$linkResults','')) (509,498)  [**View Details**](javascript:showShDetails(%22ctl00_ctl00_FindField_FindField_historyControl_ctrlPopup%22,%20%22S22%22);)  [**Edit**](http://web.b.ebscohost.com/Legacy/Views/UserControls/Ehost/) |
|  | S21 | S1 OR S2 OR S3 OR S4 | **Search modes** - Boolean/Phrase | [**View Results**](javascript:__doPostBack('ctl00$ctl00$FindField$FindField$historyControl$HistoryRepeater$ctl05$linkResults','')) (1,921,197)  [**View Details**](javascript:showShDetails(%22ctl00_ctl00_FindField_FindField_historyControl_ctrlPopup%22,%20%22S21%22);)  [**Edit**](http://web.b.ebscohost.com/Legacy/Views/UserControls/Ehost/) |
|  | S20 | transition points | **Search modes** - Boolean/Phrase | [**View Results**](javascript:__doPostBack('ctl00$ctl00$FindField$FindField$historyControl$HistoryRepeater$ctl06$linkResults','')) (4,318)  [**View Details**](javascript:showShDetails(%22ctl00_ctl00_FindField_FindField_historyControl_ctrlPopup%22,%20%22S20%22);)  [**Edit**](http://web.b.ebscohost.com/Legacy/Views/UserControls/Ehost/) |
|  | S19 | transition of care | **Search modes** - Boolean/Phrase | [**View Results**](javascript:__doPostBack('ctl00$ctl00$FindField$FindField$historyControl$HistoryRepeater$ctl07$linkResults','')) (8,490)  [**View Details**](javascript:showShDetails(%22ctl00_ctl00_FindField_FindField_historyControl_ctrlPopup%22,%20%22S19%22);)  [**Edit**](http://web.b.ebscohost.com/Legacy/Views/UserControls/Ehost/) |
|  | S18 | transition | **Search modes** - Boolean/Phrase | [**View Results**](javascript:__doPostBack('ctl00$ctl00$FindField$FindField$historyControl$HistoryRepeater$ctl08$linkResults','')) (338,132)  [**View Details**](javascript:showShDetails(%22ctl00_ctl00_FindField_FindField_historyControl_ctrlPopup%22,%20%22S18%22);)  [**Edit**](http://web.b.ebscohost.com/Legacy/Views/UserControls/Ehost/) |
|  | S17 | transfer | **Search modes** - Boolean/Phrase | [**View Results**](javascript:__doPostBack('ctl00$ctl00$FindField$FindField$historyControl$HistoryRepeater$ctl09$linkResults','')) (472,091)  [**View Details**](javascript:showShDetails(%22ctl00_ctl00_FindField_FindField_historyControl_ctrlPopup%22,%20%22S17%22);)  [**Edit**](http://web.b.ebscohost.com/Legacy/Views/UserControls/Ehost/) |
|  | S16 | discharge* | **Search modes** - Boolean/Phrase | [**View Results**](javascript:__doPostBack('ctl00$ctl00$FindField$FindField$historyControl$HistoryRepeater$ctl10$linkResults','')) (234,782)  [**View Details**](javascript:showShDetails(%22ctl00_ctl00_FindField_FindField_historyControl_ctrlPopup%22,%20%22S16%22);)  [**Edit**](http://web.b.ebscohost.com/Legacy/Views/UserControls/Ehost/) |
|  | S15 | admission* | **Search modes** - Boolean/Phrase | [**View Results**](javascript:__doPostBack('ctl00$ctl00$FindField$FindField$historyControl$HistoryRepeater$ctl11$linkResults','')) (202,000)  [**View Details**](javascript:showShDetails(%22ctl00_ctl00_FindField_FindField_historyControl_ctrlPopup%22,%20%22S15%22);)  [**Edit**](http://web.b.ebscohost.com/Legacy/Views/UserControls/Ehost/) |
|  | S14 | medication management | **Search modes** - Boolean/Phrase | [**View Results**](javascript:__doPostBack('ctl00$ctl00$FindField$FindField$historyControl$HistoryRepeater$ctl12$linkResults','')) (8,771)  [**View Details**](javascript:showShDetails(%22ctl00_ctl00_FindField_FindField_historyControl_ctrlPopup%22,%20%22S14%22);)  [**Edit**](http://web.b.ebscohost.com/Legacy/Views/UserControls/Ehost/) |
|  | S13 | medicines management | **Search modes** - Boolean/Phrase | [**View Results**](javascript:__doPostBack('ctl00$ctl00$FindField$FindField$historyControl$HistoryRepeater$ctl13$linkResults','')) (10,266)  [**View Details**](javascript:showShDetails(%22ctl00_ctl00_FindField_FindField_historyControl_ctrlPopup%22,%20%22S13%22);)  [**Edit**](http://web.b.ebscohost.com/Legacy/Views/UserControls/Ehost/) |
|  | S12 | medicines* | **Search modes** - Boolean/Phrase | [**View Results**](javascript:__doPostBack('ctl00$ctl00$FindField$FindField$historyControl$HistoryRepeater$ctl14$linkResults','')) (53,088)  [**View Details**](javascript:showShDetails(%22ctl00_ctl00_FindField_FindField_historyControl_ctrlPopup%22,%20%22S12%22);)  [**Edit**](http://web.b.ebscohost.com/Legacy/Views/UserControls/Ehost/) |
|  | S11 | medication* | **Search modes** - Boolean/Phrase | [**View Results**](javascript:__doPostBack('ctl00$ctl00$FindField$FindField$historyControl$HistoryRepeater$ctl15$linkResults','')) (303,018)  [**View Details**](javascript:showShDetails(%22ctl00_ctl00_FindField_FindField_historyControl_ctrlPopup%22,%20%22S11%22);)  [**Edit**](http://web.b.ebscohost.com/Legacy/Views/UserControls/Ehost/) |
|  | S10 | elderly* | **Search modes** - Boolean/Phrase | [**View Results**](javascript:__doPostBack('ctl00$ctl00$FindField$FindField$historyControl$HistoryRepeater$ctl16$linkResults','')) (233,409)  [**View Details**](javascript:showShDetails(%22ctl00_ctl00_FindField_FindField_historyControl_ctrlPopup%22,%20%22S10%22);)  [**Edit**](http://web.b.ebscohost.com/Legacy/Views/UserControls/Ehost/) |
|  | S9 | seniors | **Search modes** - Boolean/Phrase | [**View Results**](javascript:__doPostBack('ctl00$ctl00$FindField$FindField$historyControl$HistoryRepeater$ctl17$linkResults','')) (53,845)  [**View Details**](javascript:showShDetails(%22ctl00_ctl00_FindField_FindField_historyControl_ctrlPopup%22,%20%22S9%22);)  [**Edit**](http://web.b.ebscohost.com/Legacy/Views/UserControls/Ehost/) |
|  | S8 | geriatric* | **Search modes** - Boolean/Phrase | [**View Results**](javascript:__doPostBack('ctl00$ctl00$FindField$FindField$historyControl$HistoryRepeater$ctl18$linkResults','')) (141,420)  [**View Details**](javascript:showShDetails(%22ctl00_ctl00_FindField_FindField_historyControl_ctrlPopup%22,%20%22S8%22);)  [**Edit**](http://web.b.ebscohost.com/Legacy/Views/UserControls/Ehost/) |
|  | S7 | older adults | **Search modes** - Boolean/Phrase | [**View Results**](javascript:__doPostBack('ctl00$ctl00$FindField$FindField$historyControl$HistoryRepeater$ctl19$linkResults','')) (74,198)  [**View Details**](javascript:showShDetails(%22ctl00_ctl00_FindField_FindField_historyControl_ctrlPopup%22,%20%22S7%22);)  [**Edit**](http://web.b.ebscohost.com/Legacy/Views/UserControls/Ehost/) |
|  | S6 | older people | **Search modes** - Boolean/Phrase | [**View Results**](javascript:__doPostBack('ctl00$ctl00$FindField$FindField$historyControl$HistoryRepeater$ctl20$linkResults','')) (29,609)  [**View Details**](javascript:showShDetails(%22ctl00_ctl00_FindField_FindField_historyControl_ctrlPopup%22,%20%22S6%22);)  [**Edit**](http://web.b.ebscohost.com/Legacy/Views/UserControls/Ehost/) |
|  | S5 | older patients | **Search modes** - Boolean/Phrase | [**View Results**](javascript:__doPostBack('ctl00$ctl00$FindField$FindField$historyControl$HistoryRepeater$ctl21$linkResults','')) (81,971)  [**View Details**](javascript:showShDetails(%22ctl00_ctl00_FindField_FindField_historyControl_ctrlPopup%22,%20%22S5%22);)  [**Edit**](http://web.b.ebscohost.com/Legacy/Views/UserControls/Ehost/) |
|  | S4 | caregivers | **Search modes** - Boolean/Phrase | [**View Results**](javascript:__doPostBack('ctl00$ctl00$FindField$FindField$historyControl$HistoryRepeater$ctl22$linkResults','')) (65,329)  [**View Details**](javascript:showShDetails(%22ctl00_ctl00_FindField_FindField_historyControl_ctrlPopup%22,%20%22S4%22);)  [**Edit**](http://web.b.ebscohost.com/Legacy/Views/UserControls/Ehost/) |
|  | S3 | carers | **Search modes** - Boolean/Phrase | [**View Results**](javascript:__doPostBack('ctl00$ctl00$FindField$FindField$historyControl$HistoryRepeater$ctl23$linkResults','')) (12,629)  [**View Details**](javascript:showShDetails(%22ctl00_ctl00_FindField_FindField_historyControl_ctrlPopup%22,%20%22S3%22);)  [**Edit**](http://web.b.ebscohost.com/Legacy/Views/UserControls/Ehost/) |
|  | S2 | relatives | **Search modes** - Boolean/Phrase | [**View Results**](javascript:__doPostBack('ctl00$ctl00$FindField$FindField$historyControl$HistoryRepeater$ctl24$linkResults','')) (808,285)  [**View Details**](javascript:showShDetails(%22ctl00_ctl00_FindField_FindField_historyControl_ctrlPopup%22,%20%22S2%22);)  [**Edit**](http://web.b.ebscohost.com/Legacy/Views/UserControls/Ehost/) |
|  | S1 | family | **Search modes** - Boolean/Phrase | [**View Results**](javascript:__doPostBack('ctl00$ctl00$FindField$FindField$historyControl$HistoryRepeater$ctl25$linkResults','')) (1,133,210)  [**View Details**](javascript:showShDetails(%22ctl00_ctl00_FindField_FindField_historyControl_ctrlPopup%22,%20%22S1%22);)  [**Edit**](http://web.b.ebscohost.com/Legacy/Views/UserControls/Ehost/) |
